# Intraocular pressure changes before and after a femtosecond laser procedure for cataract surgery

**DOI:** 10.1038/s41598-024-55961-2

**Published:** 2024-04-19

**Authors:** Ho Seok Chung, Hun Lee, So young Park, Chan Hong Min, Mose Kim, Jae Yong Kim, Hungwon Tchah

**Affiliations:** 1 Department of Ophthalmology, Asan Medical Center, University of Ulsan College of Medicine, Seoul, Korea; 2 Department of Ophthalmology, Kim’s Eye Hospital, Myung-Gok Eye Research Institute, Konyang University College of Medicine, 136, Yeongsin-ro, Yeongdeungpo-gu, Seoul, 07031 South Korea

**Keywords:** Eye manifestations, Eye diseases

## Abstract

This study aimed to evaluate the changes in intraocular pressure (IOP) before and after femtosecond laser capsulorhexis and lens fragmentation for cataract surgery. We measured the IOP before, immediately, 30 min, and 1 h after the laser procedure in 47 eyes of 47 patients who underwent the femtosecond laser procedure. The mean IOP was 17.51 ± 3.28 mmHg, 30.23 ± 6.70 mmHg, 17.96 ± 3.75 mmHg, and 21.77 ± 5.88 mmHg before, immediately after, 30 min after, and 1 h after the laser procedure, respectively. The mean IOP significantly increased immediately (adjusted P < 0.001) and 1 h (adjusted P = 0.001) after the laser procedure compared with the pre-laser IOP. The mean IOP at 30 min after the laser procedure was significantly lower than that immediately after the procedure (adjusted P < 0.001). However, the IOP 1 h after the laser procedure became higher than that 30 min after the laser procedure. Additionally, the IOP 1 h after the laser procedure was positively correlated with the baseline IOP and negatively correlated with the axial length. In conclusion, this study demonstrated that cataract surgery should be commenced within 30 min after the femtosecond laser procedure to ensure a safe cataract surgery that reduces the risk of increased intraocular pressure.

## Introduction

The femtosecond laser has recently been used in cataract surgery for corneal incision, continuous curvilinear capsulorhexis (CCC), and lens fragmentation, as well as in astigmatism correction^1–5^. The advantages of the femtosecond laser include accurate CCC with good intraocular lens (IOL) centration, minimal loss of endothelium, and a short phacoemulsification time^6–11^. Femtosecond laser-assisted cataract surgery (FLACS) had an advantage over conventional methods in minimizing endothelial cell loss after cataract surgery^12^.

Contrastingly, complications of using the femtosecond laser, such as suction loss, conjunctival injection, conjunctival hemorrhage, CCC tag, CCC tear, and miosis, have also been reported^13^. In addition, intraocular pressure (IOP) elevation occurs in porcine eyes when applying the suction ring and vacuum of the femtosecond laser^14–16^. Preoperative IOP elevation during cataract surgery could be a risk factor for suprachoroidal hemorrhage, a rare but significant complication of cataract surgery^17^. Therefore, IOP evaluation is crucial to preventing this serious complication during cataract surgery when using the femtosecond laser.

Several studies have reported changes in the IOP before and after performing femtosecond laser procedures in humans^18–22^. However, to the best of our knowledge, no published studies have evaluated the changes in IOP at certain intervals before and after femtosecond laser procedures. Because the femtosecond laser device is often located separately because of the lack of space in the operating room, monitoring the changes in IOP over time between the surgery and the laser procedure is necessary to ensure safety. Therefore, this study aimed to evaluate the changes in IOP before, immediately after, 30 min after, and 1 h after a femtosecond laser procedure.

## Results

In this study, 47 eyes of 47 patients were included. Table 1 shows the demographic and clinical characteristics of the study participants. The mean nuclear sclerosis grade was 3.67 ± 0.94, and the preoperative corneal astigmatism, based on the autokeratometry, was 0.92 ± 0.85 D. Based on slit-lamp microscopy examinations, the nuclear sclerosis grades (graded according to the LOCS III) were grade 2 in 17.0% (8 eyes), grade 3 in 29.8% (14 eyes), grade 4 in 38.3% (18 eyes), and grade 5 in 14.9% (7 eyes) of the eyes. The laser energy used for CCC was 0.3 or 0.4 J, and that for lens fragmentation was approximately 5.0 to 11.5 J, according to the laser time. Arcuate incision (AI) was performed according to the patient’s corneal astigmatism, and the average laser energy for AI was 1.47 ± 1.01 J.

**Table 1:** Demographic and clinical characteristics of the study participants.

Parameter	
Number of eyes/patients	47/47
Sex (male/female)	17/30
Age (years)	65.8 ± 5.7 (range: 55 to 76)
Nuclear sclerosis grade	3.67 ± 0.94
Mean corneal astigmatism (D)	0.92 ± 0.85
Preoperative sphere (D)	− 3.71 ± 4.86
Preoperative cylinder (D)	1.39 ± 1.07
Preoperative pachymetry (µm)	576.85 ± 33.83
Preoperative axial length (mm)	24.32 ± 1.83
Preoperative anterior chamber depth (mm)	2.73 ± 0.45
Total femtosecond laser energy (J)	8.50 ± 1.74

The results are reported as means ± standard deviations. *D* diopters.

**Figure 1:**
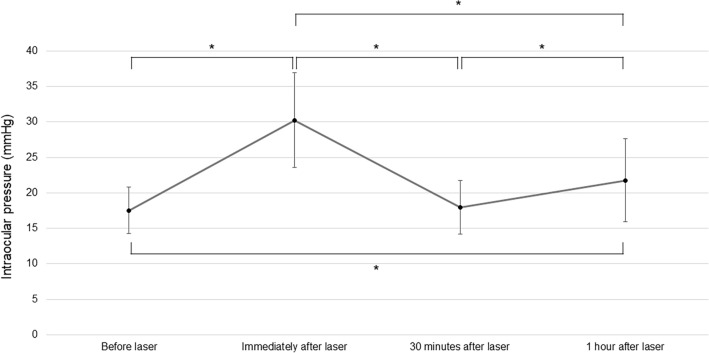
The mean intraocular pressure before and after the femtosecond laser procedure Error bars represent the standard deviation of the mean (*P < 0.05, Friedman test with post hoc Bonferroni correction).

**Figure 2:**
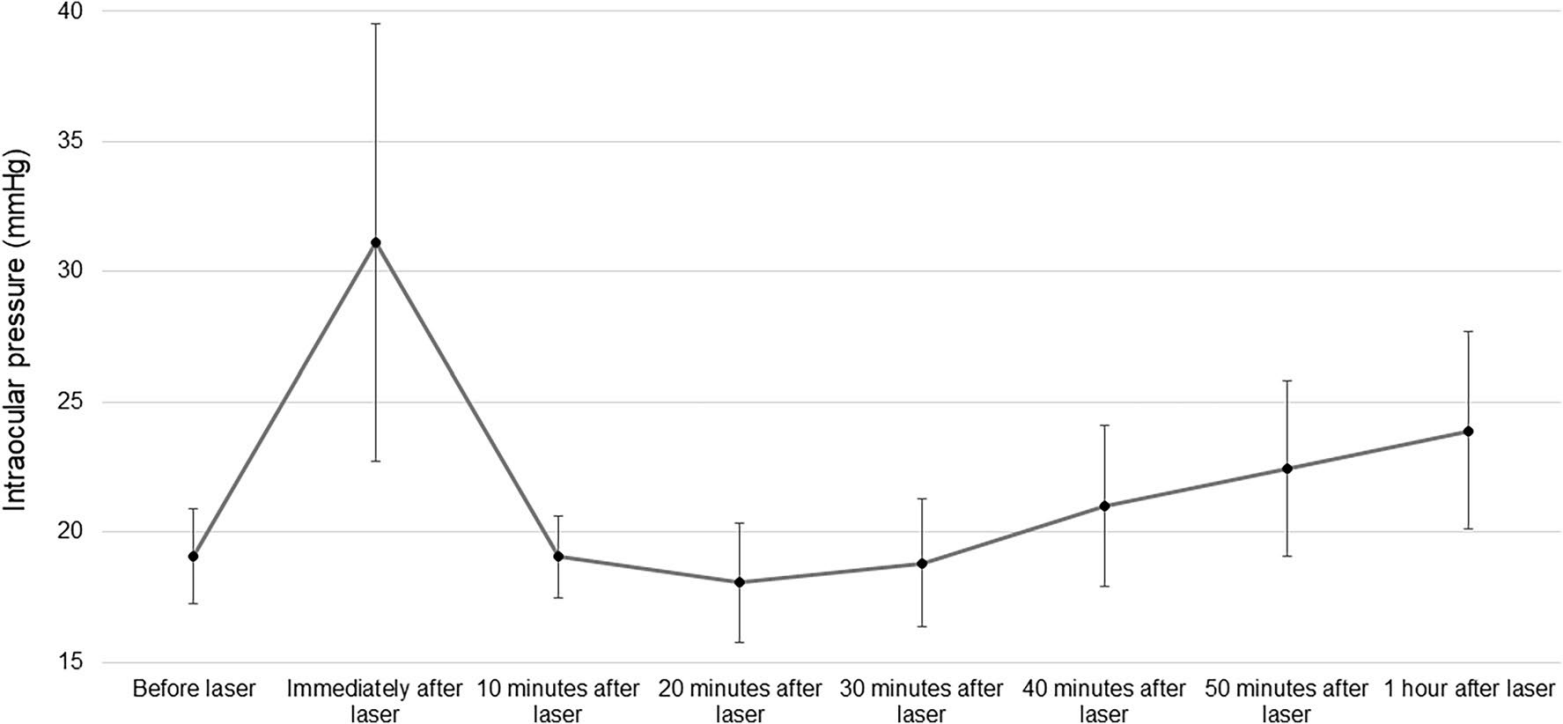
The mean intraocular pressure before femtosecond laser and every 10 min until 1 h after the femtosecond laser procedure for 16 eyes. Error bars represent the standard deviation of the mean.

The mean IOPs before, immediately after, at 30 min after, and at 1 h after the laser procedure were 17.51 ± 3.28 mmHg, 30.23 ± 6.70 mmHg, 17.96 ± 3.75 mmHg, and 21.77 ± 5.88 mmHg, respectively (Table 2). Compared with the IOP before the laser procedure, the mean IOP immediately after (adjusted P < 0.001) and at 1 h after the laser procedure (adjusted P = 0.001) was significantly increased. In contrast, the mean IOP 30 min after the laser procedure (adjusted P < 0.001) was significantly decreased compared with that immediately after the laser procedure. Furthermore, there was a significant increase in the mean IOP at 1 h after the laser procedure (adjusted P < 0.001) compared with the IOP at 30 min after the laser procedure. The mean IOP before and 30 min after the laser procedure was not significantly different (Fig. 1). The mean IOPs per 10 min for up to 1 h after the femtosecond laser procedure for 16 eyes are shown in Table 3 and Fig. 2. The IOP remained similar from 10 to 30 min after the femtosecond laser procedure and then gradually increased until 1 h after the femtosecond laser procedure.

**Table 2:** Changes in the intraocular pressure before and after the femtosecond laser procedure (N = 47).

	Before the laser procedure	Immediately after the laser procedure	30 min after the laser procedure	1 h after the laser procedure	*P* value*
IOP (mmHg)					
Mean	17.51	30.23	17.96	21.77	< 0.001
SD	3.28	6.70	3.75	5.88	
Range	10 to 25	20 to 49	10 to 28	11 to 44	

*IOP* intraocular pressure, *SD* standard deviation, *Friedman test.

**Table 3:** Changes in the intraocular pressure before and after the femtosecond laser procedure (N = 16).

	Before the laser procedure	Immediately after the laser procedure	10 min after the laser procedure	20 min after the laser procedure	30 min after the laser procedure	40 min after the laser procedure	50 min after the laser procedure	1 h after the laser procedure
IOP (mmHg)								
Mean	19.06	31.13	19.06	18.06	18.81	21.00	22.44	23.88
SD	1.84	8.39	1.57	2.29	2.48	3.08	3.37	3.79
Range	16 to 22	20 to 49	15 to 21	12 to 22	15 to 24	16 to 25	17 to 27	17 to 31

*IOP* intraocular pressure, *SD* standard deviation.

Regarding the regression analysis, data for 47 eyes and 47 patients’ personal data were analyzed. In the simple regression analysis, age (R^2^ = 0.008, P = 0.554), nuclear sclerosis grade (R^2^ = 0.000, P = 0.967), sex (R^2^ = 0.017, P = 0.385), anterior chamber depth (R^2^ = 0.032, P = 0.229), and laser energy (R^2^ = 0.007, P = 0.567) were not independent factors affecting the IOP at 1 h after the laser procedure. The factors that were significant in the simple regression were the axial length (R^2^ = 0.128, P = 0.014) and the baseline IOP (R^2^ = 0.148, P = 0.008). In the multiple regression analyses for these factors, the IOP at 1 h after the laser procedure was positively correlated with the baseline IOP (B = 0.637, P = 0.011) and negatively correlated with the axial length (B = -1.031, P = 0.019) (Table 4). In addition, the mean IOP at each time point between the low nuclear sclerosis grade (grades 2 and 3) and high nuclear sclerosis grade (grades 4 and 5) groups (Table 5) was not significantly different. Moreover, there was no significant difference in the mean IOP at each time point between the low total laser energy (mean energy less than 8.45 J) and high total laser energy (mean energy greater than 8.45 J) groups (Table 6).

**Table 4:** Multiple regression analysis of factors affecting the intraocular pressure at 1 h after the femtosecond laser procedure.

Factors	IOP at 1 h after the laser procedure			*P*	VIF
	B	SE	β		
Axial length	− 1.031	0.423	− 0.320	0.019	1.012
IOP before the procedure	0.637	0.239	0.351	0.011	1.012
F (*P* value)	7.318 (0.002)				
Durbin–Watson	2.190				
R^2^	0.250				

*IOP* intraocular pressure.

**Table 5:** Comparison of the intraocular pressure before and after the femtosecond laser procedure between the low-grade cataract and high-grade cataract groups.

Intraocular pressure (mmHg)	Low nuclear sclerosis grade (N = 22)	High nuclear sclerosis grade (N = 25)	*P* value
Before the laser procedure	17.64 ± 3.44	17.40 ± 3.20	0.864
Immediately after the laser procedure	29.91 ± 8.02	30.52 ± 5.44	0.258
30 min after the laser procedure	18.32 ± 3.43	17.64 ± 4.05	0.622
1 h after the laser procedure	21.26 ± 4.49	22.08 ± 7.10	0.661

The results are reported as means ± standard deviations.

**Table 6:** Comparison of the intraocular pressure before and after the femtosecond laser procedure between the low total laser energy and high total laser energy groups.

Intraocular pressure (mmHg)	Low total laser energy (J) (N = 25)	High total laser energy (J) (N = 22)	*P* value
Before the laser procedure	17.75 ± 2.83	17.26 ± 3.74	0.548
Immediately after the laser procedure	29.50 ± 6.81	31.00 ± 6.6	0.551
30 min after the laser procedure	18.21 ± 3.80	17.70 ± 3.76	0.645
1 h after the laser procedure	21.04 ± 4.79	22.52 ± 6.87	0.974

The results are reported as means ± standard deviations.

## Discussion

In the present study, we evaluated the changes in IOP before and after a femtosecond laser procedure and thus demonstrated that the IOP significantly increased immediately after the femtosecond laser procedure, normalized at 30 min after the laser procedure, but increased again at 1 h after the femtosecond laser procedure. FLACS has been reported to make cataract surgery easier and IOL centration more precise by ensuring a precise CCC. Therefore, a high quality of vision can be expected after FLACS^24^. In addition, FLACS reduces the phacoemulsification power and effective phacoemulsification time by preoperative lens fragmentation, accompanied by minimal loss of endothelial cells^7–10^.

The interface of the femtosecond laser and the cornea of the patient should be strongly attached by a vacuum before applying the femtosecond laser. Moreover, CCC can lead to the release of fragmented lens materials into the anterior chamber after a femtosecond laser procedure. Few studies have investigated the effects of these situations on ocular parameters such as the IOP. In experimental researches, IOP was increased in porcine eyes during the suction ring application of the femtosecond laser device^14–16^. In addition, during the vacuum application, the anterior and posterior structures of the eye could be changed^25^. Consequently, binocular vitreous detachment, retinal hemorrhage, and changes in ocular blood supply have been reported^26–28^. Although changes in the IOP before and after the femtosecond laser procedure have been reported, no published report has evaluated the changes in the IOP at certain time intervals before and after the laser procedure in humans^18–22^. Therefore, we sought to evaluate the IOP before and after a laser procedure at 30-min intervals for up to 1 h to ensure the safety of the femtosecond laser procedure.

Overall, in the present study, IOP significantly changed with time; the IOP immediately after the laser procedure was significantly higher than that before the laser procedure, and the IOP subsequently decreased significantly. However, it increased again over time. One study reported that increased IOP normalized immediately after the removal of the interface of the femtosecond laser^22^. However, in this study, the IOP remained higher than that before the laser procedure, even after removing the interface of the femtosecond laser. This result is similar to those of previous studies, which reported that the IOPs immediately after laser procedures were significantly higher than those before laser procedures, even after suction ring removal^18–21^. We hypothesized that this difference in changes in the IOP may be due to differences in femtosecond laser systems. Baig et al. used the Victus platform (Bausch & Lomb, Germany), which uses both the fluid-filled interface and the curved contact lens docking system during FLACS in two steps^22^. However, in all the others, as well as in our study, we used the Catalys Precision system with only a liquid optics interface^18–20^. De Giacinato et al. compared IOP changes between the Catalys Precision system and LenSx Laser (Alcon Laboratories, Inc., Fort Worth, TX, USA) with a curved interface and soft contact lens. The results showed a marked increase in IOP with the Catalys system than with the LenSx Laser^21^. The pressure of the suction ring and the vacuum force of the liquid optics interface probably contributed to the significant IOP increase immediately after the laser procedure.

In our study, the IOP decreased to a value similar to the pre-laser IOP 30 min after the laser procedure. This may have been caused by an aqueous humor turnover rate of approximately 1.0–1.5% per minute^29^. The number of tiny lens particles or macrophages released into the anterior chamber and inflammatory cytokines may have been diluted by the replacement of the intraocular volume^29^. The mean IOP at 1 h after the laser procedure was not as high as that immediately after the laser procedure, but the IOP increased significantly compared to that before and that at 30 min after the laser procedure. We suggest that fragmented lens particles may have blocked the trabecular meshwork, and CO_2_ bubbles produced after the femtosecond laser procedure may have increased the anterior chamber volume, both leading to the increased IOP. Based on the pathophysiology of phacolytic glaucoma, tiny lens particles or macrophages, even though invisible, could temporarily enter the anterior chamber, leading to an increased IOP^30,31^. In addition, inflammatory cytokines that were upregulated by the laser procedure could induce acute inflammation of the trabecular meshwork, leading to its blockage^32,33^.

We also hypothesized that increased IOP at 1 h after the femtosecond laser procedure may be affected by age, nuclear sclerosis grade, axial length, the IOP before the laser procedure, and the total laser energy. Among these, the IOP before the laser procedure was positively correlated with the IOP at 1 h after the laser procedure, and axial length was negatively correlated with the IOP at 1 h after the laser procedure. Previous studies also reported that an increase in the IOP during a laser procedure in porcine eyes was more correlated with the baseline IOP than the level of applied vacuum^16,34^. Additionally, we found a negative correlation between axial length and the IOP 1 h after the femtosecond laser procedure. This may be because a patient with a long axial length has a large eyeball volume, and the large volume plays a role in buffering the force exerted by suction during the laser procedure. Our results suggest that surgeons should look out for increases in the IOP of patients with a high preoperative IOP and a short axial length during laser procedures. Further evaluation with more patients is required to clarify this.

We measured the IOP every 10 min until 1 h after the laser procedure in our study. The IOP was usually lowered to the pre-laser level 10 min after the procedure and maintained at the same level until 30 min after the laser procedure. However, considering that the reduced IOP increased again 1 h after the laser procedure, we recommend that cataract surgery be performed within 30 min after a femtosecond laser procedure, during the time of stable IOP. Although the average IOP at 1 h after the femtosecond laser procedure was 21.77 mmHg, which is not high enough to cause optic nerve damage, the IOP ranged from 11 to 44 mmHg. Moreover, there were 12 eyes (26%) where the IOP was 25 mmHg or higher. Furthermore, even transient increases in the IOP can cause optic nerve damages and serious complications^17,35,36^. Therefore, we suggest focusing on those eyes with a tendency to develop high IOP rather than on their average IOP value. Some studies recommend using topical NSAIDs to reduce the inflammatory cytokine reflex, including prostaglandins, which can induce miosis^33,35,37^. Further research that investigates the ideal timing of performing cataract surgery after a femtosecond laser procedure is required to ensure patient safety and procedural efficiency.

We used Tono-Pen AVIA (Reichert Inc., Buffalo, NY, USA) to measure the IOP in the supine position, as it was necessary to measure the IOP quickly while maintaining the supine position. Because an intrastromal arcuate keratotomy was performed, there was no change in the corneal surface. Furthermore, it can be assumed that corneal edema resulting from the femtosecond laser procedure may cause a slight error in the IOP measurement. However, in a similar situation, a previous study showed that the IOP measurement obtained with the Tono-Pen was more accurate than that obtained with the Goldmann applanation tonometry owing to its small contact area with the corneal surface^38^. Therefore, we believe the measurement to be comparatively valid. However, because this is a hand-held device, the effect of instability should also be considered.

This study is limited by its relatively small number of patients and its retrospective design. IOP can be affected by the patient's anxiety about surgery and emotional stress, which were not included in the analysis in this study. In addition, our results are only applicable to situations where the same device, the Catalys system, is used; the study of IOP changes over time in cases where other femtosecond laser devices are used is required. Further prospective studies with a larger sample size and serial time interval IOP measurements in various femtosecond laser systems are required.

In summary, this is the first study that has serially evaluated IOP changes after a femtosecond laser procedure for cataract surgery. The mean IOP significantly increased immediately after the femtosecond laser procedure and then decreased significantly after 30 min. The IOP increased again 1 h after the laser procedure. The IOP 30 min after the laser procedure was not significantly different from that before the laser procedure. Therefore, we recommend that cataract surgery be performed within 30 min after a femtosecond laser procedure to ensure a safe and successful cataract surgery.

## Methods

We conducted this observational case series with the approval of the Institutional Review Board of the Asan Medical Center and the University of Ulsan College of Medicine, Seoul, South Korea (2019–0774). Informed consent was obtained from all patients, and the study adhered to the tenets of the Declaration of Helsinki and followed good clinical practice guidelines. The study was registered with WHO/ICTRP, registration number: KCT0004710.

This study included patients who underwent femtosecond laser-assisted cataract surgery (FLACS) performed by the same surgeon at the Asan Medical Center. Patients who met the following criteria were included: (1) aged > 18 years; (2) have pre-existing corneal astigmatism of less than + 2.50 D; and (3) have a visual acuity of greater than 0.1 logMAR as measured using a potential acuity meter. Patients were excluded from the analyses if they had (1) optical opacities or pathology detected on slit-lamp examination; (2) previous corneal surgeries; (3) ocular trauma; (4) intraocular surgery; (5) severe dry eyes; (6) corneal disease; (7) glaucoma and ocular hypertension; (8) ocular infection; or (9) collagen vascular disease or other autoimmune diseases.

Preoperative assessments included autokeratometry (Canon R-50, Canon USA Inc., Huntington, NY, USA), slit-lamp examinations (Haag-Streit, Gartenstadtstrasse, Köniz, Switzerland), and corneal topography (Orbscan, Bausch & Lomb, Rochester, NY, USA). The nuclear sclerosis grade was measured based on the Lens Opacities Classification System (LOCS) III during slit-lamp examinations^23^. The anterior chamber depth was measured using anterior segment optical coherence tomography (Visante OCT, Carl Zeiss Meditec, Germany). Anterior chamber depth was defined as the vertical distance from the internal border of the central corneal endothelium to the line connecting both iris recesses. The IOP was measured using Tono-Pen AVIA (Reichert Inc., Buffalo, NY, USA) in the supine position before, immediately after, 30 min after, and 1 h after the femtosecond laser procedure. Each measurement was repeated three times, and the average was taken.

FLACS was performed using the Catalys Precision Laser System (Johnson & Johnson, Milpitas, CA, USA) for CCC, cataract fragmentation, and intrastromal arcuate keratotomy. The same method and parameters were used for CCC and lens fragmentation despite differences in the nuclear sclerosis grade of each patient. An AI was performed to correct astigmatism for all patients based on the degree of corneal astigmatism of each patient. For CCC, the “Scanned Capsule” mode was used, the diameter was 4.9 mm, the horizontal spot spacing was 5 µm, and the vertical spot spacing was 10 µm. Regarding lens fragmentation, the “Quadrants Softened” mode was used, the segmentation repetitions were 5, the horizontal spot spacing was 10 µm, and the vertical spot spacing was 40 µm. Regarding the AI, the “intrastromal” mode was used, the horizontal spot spacing was 5 µm, and the vertical spot spacing was 10 µm. In addition, 0.1 J of energy was used for each 5 degrees of the arc length.

Cataract surgery commenced 1 h after the femtosecond laser procedure. The Whitestar Signature Pro System (Johnson & Johnson, Milpitas, CA, USA) was used for the cataract surgery. The cornea was manually incised with an incision knife. After the insertion of the ocular viscoelastic devices (OVD), hydrodissection was performed through the CCC margin that was already made by the femtosecond laser. Fragmented lens materials were removed using a phacoemulsifier, after which the cortex was removed, the IOL was implanted, and the OVD was removed. The surgery ended after corneal hydration was performed. A single course of a ketorolac 0.45%, proparacaine 0.5%, and mixture of phenylephrine 0.5% and tropicamide 0.5% were administered 30 min before the femtosecond laser procedure. Topical antibiotics, steroids, and non-steroidal anti-inflammatory drugs were used until 1 month postoperatively.

### Statistical analysis

A Shapiro–Wilk test was used to assess the distribution of numerical data. The Friedman test with post-hoc Bonferroni correction was used to investigate changes in IOP over time within a group of patients. The power analysis of the Friedman test showed that the statistical power of this study with 47 subjects was 99% and that sufficient participants were included. The Mann–Whitney test was used to compare the IOP between two different nuclear sclerosis grade groups and between two different total laser energy groups. A simple regression analysis was conducted to evaluate the correlation of age, nuclear sclerosis grade, sex, anterior chamber depth, laser energy, axial length, baseline IOP with IOP at 1 h after the femtosecond laser procedure. Multiple regression analyses were performed for factors with a P value of less than 0.05 in the simple regression analysis. There were no missing values among the variables included in the analysis. All statistical analyses were performed using SPSS 25.0 (IBM Corp., Armonk, NY, USA). A P value < 0.05 was considered statistically significant.

### Ethics declarations

This study was approved by the Institutional Review Board of the Asan Medical Center and the University of Ulsan College of Medicine, Seoul, South Korea (2019–0774). Informed consent was obtained from all patients, and the study adhered to the tenets of the Declaration of Helsinki and followed good clinical practice guidelines.

## Data Availability

The data used in this study are available from the corresponding author upon request.
